# The effects of low pH on the taste and amino acid composition of tiger shrimp

**DOI:** 10.1038/s41598-021-00612-z

**Published:** 2021-10-27

**Authors:** Hsueh-Han Hsieh, Veran Weerathunga, W. Sanjaya Weerakkody, Wei-Jen Huang, François L. L. Muller, Mark C. Benfield, Chin-Chang Hung

**Affiliations:** 1 Department of Oceanography, National Sun Yat-Sen University, Kaohsiung, 80424 Taiwan, ROC; 2 Department of Fisheries and Aquaculture, Faculty of Fisheries and Marine Sciences and Technology, University of Ruhuna, Matara, Sri Lanka; 3 Department of Oceanography and Coastal Sciences, Louisiana State University, Baton Rouge, 70803 LA USA

**Keywords:** Environmental impact, Biooceanography

## Abstract

Recent research has revealed that shrimp sensory quality may be affected by ocean acidification but we do not exactly know why. Here we conducted controlled pH exposure experiments on adult tiger shrimp, which were kept in 1000-L tanks continuously supplied with coastal seawater. We compared survival rate, carapace properties and flesh sensory properties and amino acid composition of shrimp exposed to pH 7.5 and pH 8.0 treatments for 28 days. Shrimp reared at pH 7.5 had a lower amino acid content (17.6% w/w) than those reared at pH 8.0 (19.5% w/w). Interestingly, the amino acids responsible for the umami taste, i.e. glutamate and aspartic acid, were present at significantly lower levels in the pH 7.5 than the pH 8.0 shrimp, and the pH 7.5 shrimp were also rated as less desirable in a blind quality test by 40 volunteer assessors. These results indicate that tiger shrimp may become less palatable in the future due to a lower production of some amino acids. Finally, tiger shrimp also had a lower survival rate over 28 days at pH 7.5 than at pH 8.0 (73% vs. 81%) suggesting that ocean acidification may affect both the quality and quantity of future shrimp resources.

## Introduction

Among growing concern about environmental changes caused by the increasing partial pressure of CO_2_ (*p*CO_2_) in the atmosphere, ocean acidification (OA) has become a key issue that has been investigated extensively during the past few decades. According to current projections, continued uptake of atmospheric CO_2_ by the ocean may lead to a decrease in the average pH of open ocean surface water of 0.4 to 0.5 by the end of the twenty-first century^1^. Beyond those predictions, coastal waters seem to be acidifying at much faster rates than the open ocean^2,3^. Moreover, the decreasing pH trend is superimposed on a very large natural pH variability in coastal waters. Critical insights into the impacts of ocean acidification (OA) may be gained by investigating its effects on the physiology of marine organisms and how seafood may be affected^4^.

A large body of evidence is available on the negative impacts of projected OA on the survival, growth, calcification, immune responses, and reproduction of marine organisms. Only recently, however, have a very small number of experimental studies attempted to estimate the possible socio-economic consequences of OA acidification due to the altered quality of seafood^5–7^. One notable such study revealed that culturing shrimp in acidified seawater negatively affected their flavor ^5^. By contrast, a study by Lemasson et al.^6^ found that high *p*CO_2_ (≈ 1000 ppm), low pH (≈ 7.63), and high temperature (20 °C) conditions did not significantly affect the aroma, appearance, or taste of the Pacific oyster *Crassostrea gigas*. The flavor of seafood comes mainly from amino acids, nucleotides, sugars and mineral salts^8^. Amino acids in particular are thought to modulate the sensory qualities of shrimp, including sweetness, bitterness and umami taste. In humans, amino acids activate specific taste receptors^9^ as well as nutrient demand. As such, the concentrations and relative proportions of amino acids in shrimp and other seafood can have important repercussions for consumers and seafood producers. San Martin et al.^7^ developed a model to test how OA could impact on the taste of seafood and its appeal to consumers. They found that the attributes of mussels that are affected by OA also tend to determine consumers’ preferences and that people would only be prepared to buy mussels affected by OA if they were 52% cheaper than they are at present.

Globally, seafood from capture fisheries and marine aquaculture contribute to 16% of animal protein consumed by humans^7^. Seafood consumption per capita doubled between 1960 (≈ 10 kg) and 2014 (≈ 20 kg)^10,11^. Total seafood production by both mariculture and capture fishery was 115.2 million tons in 2018^12^. The world population could be as high as 12 billion by 2100^13^. Hence, the demand for seafood will inevitably increase. Given this ever-increasing demand, and given the mixed results from previous studies concerning future seafood quality, there is a pressing need to expand our understanding of the effects of acidification on the quality of seafood products.

Here we exposed tiger shrimps (*Penaeus monodon*) to pH conditions covering the present (pH 8.0) and near future (pH 7.5) average pH conditions in coastal ecosystems. The tiger shrimp (*Penaeus monodon*) is a key species in the shrimp industry worldwide and its global trade is worth US$10 billion. Its annual production is about 1.5 million tons and its texture and flavor are rated as desirable and very good by consumers worldwide^11,14^. Tiger shrimp live in brackish, estuarine (juveniles), and marine (adults) environments that extend from Africa to southern Asia^15^. Increasingly, tiger shrimp are farmed in the coastal and wetland regions of south Asian countries. These coastal waters are currently affected by rapid ocean acidification which may threaten or affect the health, production rates, and meat quality of future tiger shrimp.

We evaluated the changes of survival, growth, amino acid concentration of flesh, and sensory quality of meat in tiger shrimps under low pH conditions compared to high pH conditions. A blind tasting test was conducted to detect sensory changes while amino acid concentrations were used as a proxy to trace the changes. Different amino acids have different dominant taste qualities. Basically, threonine, serine, glycine, alanine, arginine, and proline are responsible for sweetness, valine, leucine, tyrosine and phenylalanine for bitterness, while glutamic acid and aspartic acids deliver the umami taste^16–19^. We hypothesized that observed differences in the taste of shrimp would be explained by the concentrations of the above amino acids.

## Results

**Figure 1:**
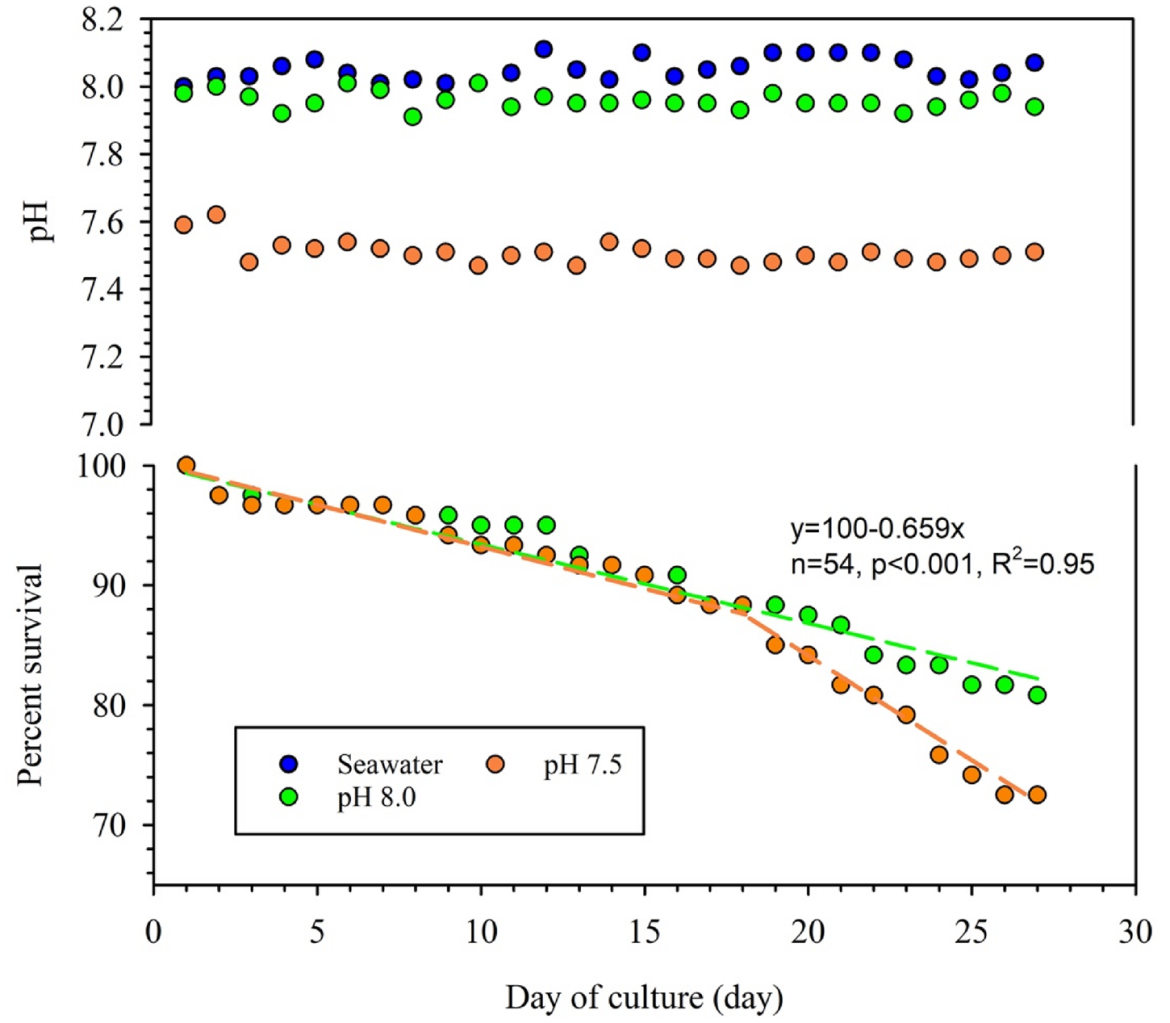
Time courses of pH and percent survival of shrimp during the culture periods Blue circles = source seawater; green circles = seawater in pH 8.0 tanks; orange circles = seawater in pH 7.5 tanks. The green and orange broken lines are regression lines representing the percentage of surviving shrimp in the pH 8.0 and pH 7.5 tanks, respectively.

Mean pH values measured in the target pH 8.0 and pH 7.5 experimental tanks throughout the experiment were 7.96 ± 0.03 and 7.51 ± 0.04, respectively. Percent of survival in pH 8.0 and pH 7.5 after 28 days were 80.8% and 72.5%, respectively. The pH 8.0 tanks declined linearly with time over the entire 28-day period. The mean mortality rate was 0.7% per day, where % refers to the initial, total number of shrimp, not the total number of shrimp on the day of measurement. In the pH 7.5 tanks, shrimp populations declined at the same rate as in the pH 8.0 tanks until day 18. From day 18 to 28, however, mortality occurred at a faster-although still linear-rate of 2.0% per day (Fig. 1).

**Figure 2:**
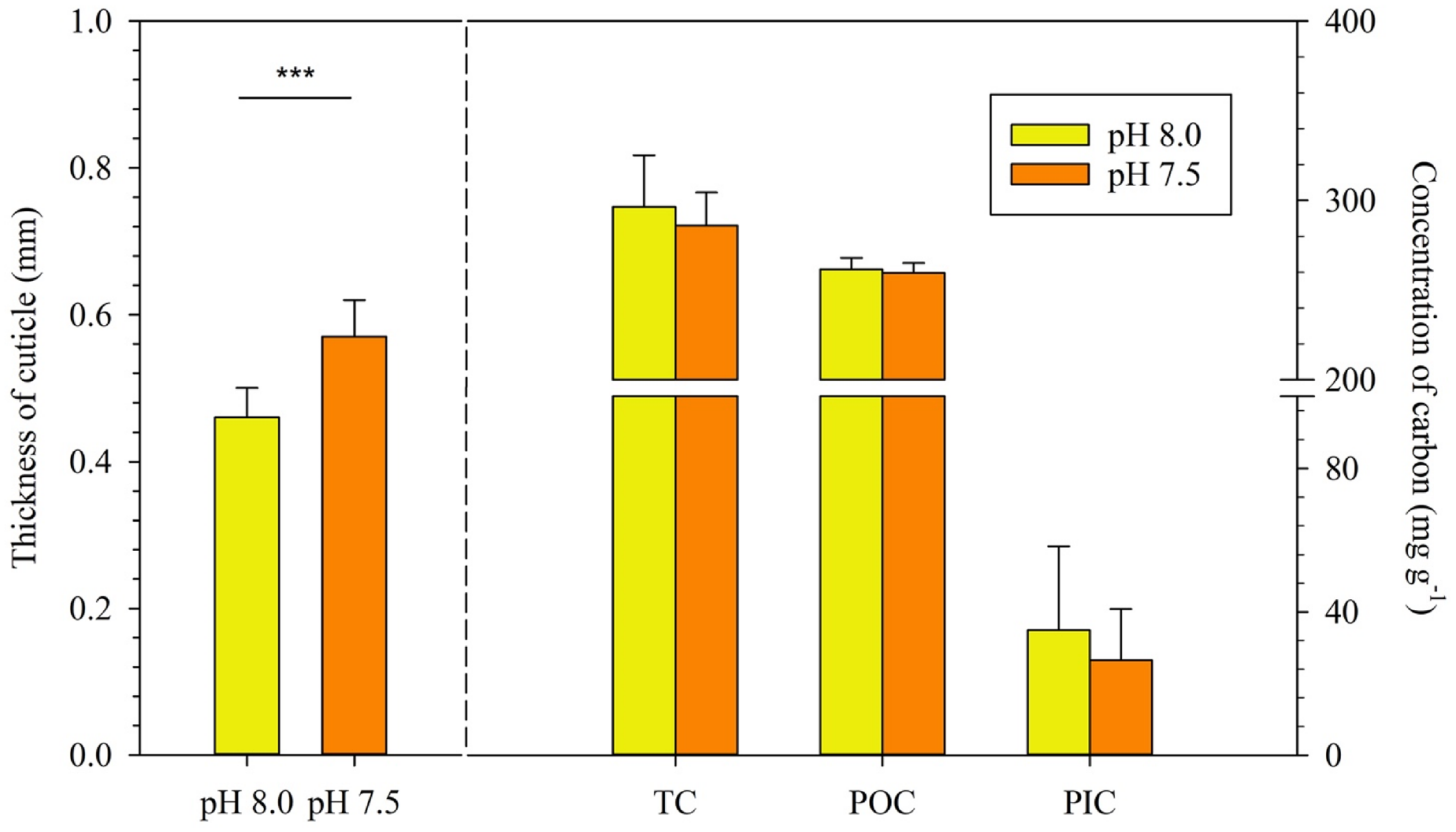
Thickness and carbon content of cuticle in pH 8.0 and pH 7.5 treatments. Left: Thickness of cuticle, where the height of each column represents mean ± S.E. (n = 30) and asterisks indicate a significant difference at *p* < 0.001. Right: Concentration of total carbon (TC), particulate organic carbon (POC) and particulate inorganic carbon (PIC) in shrimp cuticle, where column heights are means ± S.E (n = 6). No significant differences were found between the values of TC, POC or PIC measured in the pH 8.0 and pH 7.5 treatments.

Interestingly, the average carapace thickness of shrimp at the end of the pH 7.5 treatment was 0.57 ± 0.28 mm, i.e., significantly higher than the carapace thickness of 0.46 ± 0.28 mm measured after the pH 8.0 treatment (n = 30, *p* < 0.001, Fig. 2). Total, organic and inorganic carbon contents in shrimp carapace were 297, 262 and 35 mg g^−1^ at pH 8.0, versus 276, 258 and 18 mg g^−1^ at pH 7.5. The results of unpaired *t*-test showed that there was no significant difference among the carbon contents measured after the pH 8.0 and pH 7.5 treatments (Fig. 2).

**Figure 3:**
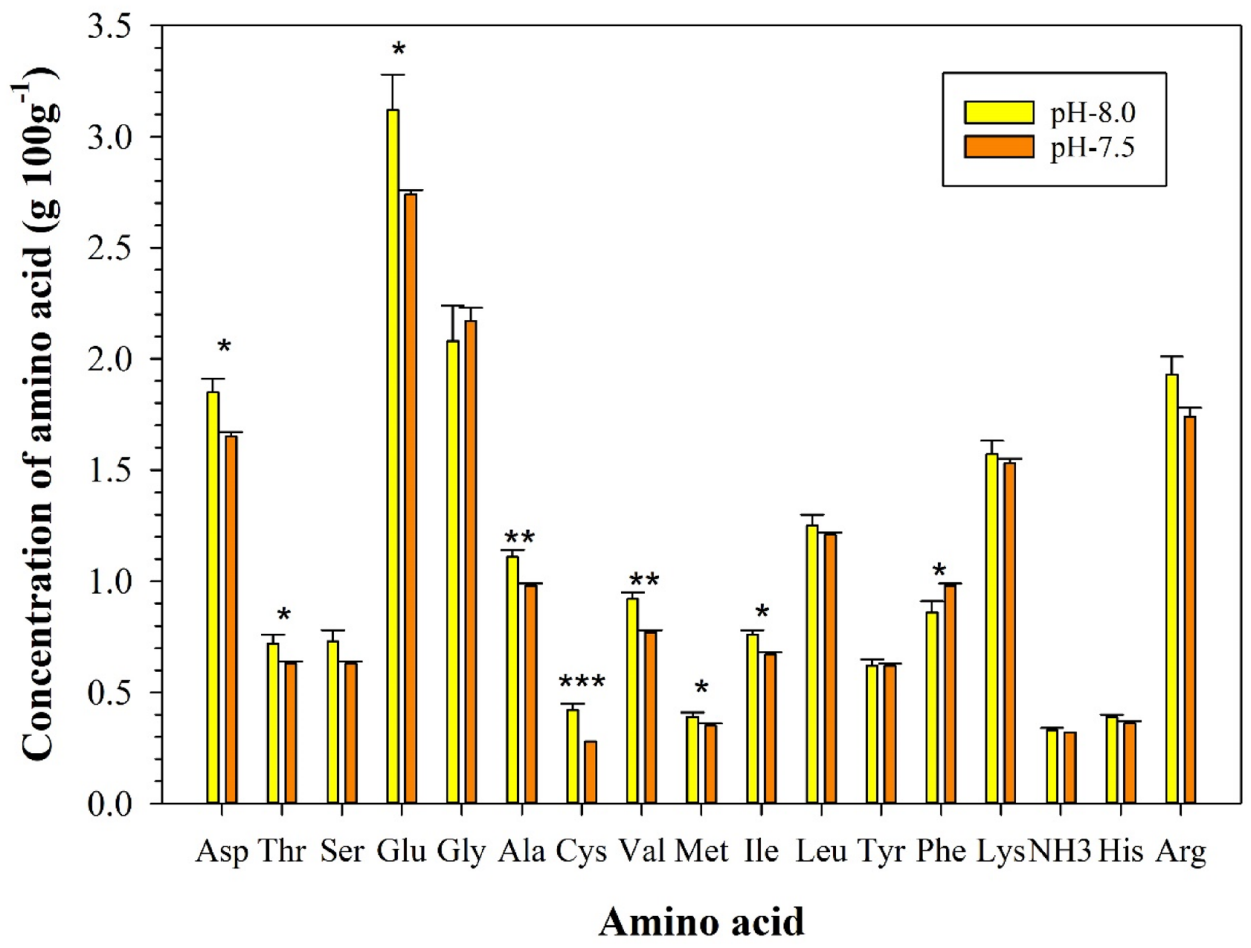
Concentrations of amino acids in the muscle of tiger shrimp at two different pH treatments (8.0 and 7.5). Values are means ± S.E (n = 6). Asterisk: statistically significant (*p < 0.05, **p < 0.01, ***p < 0.001). Asp (Aspartic acid), Thr (Threonine), Ser (Serine), Glu (Glutamic acid), Gly (Glycine), Ala (Alanine), Cys (Cysteine), Val (Valine), Met (Methionine), Ile (Isoleucine), Leu (Leucine), Tyr (Tyrosine), Phe (Phenylalanine), Lys (Lysine), His (Histidine), Arg (Arginine).

The total amino acid content in shrimp flesh was 19.5 ± 0.69 g 100 g^−1^ at pH 8.0 and 17.65 ± 0.13 g 100 g^−1^ at pH 7.5, i.e. showing no significant difference between the two treatments (n = 6, *p* = 0.07) (Fig. 3 and Table S1 showing individual amino acid composition). Among the amino acids, asparagine, threonine, glutamic acid, alanine, cysteine, valine, methionine, isoleucine were present at significantly higher levels in the pH 8.0 than the pH 7.5 treatment (n = 6, *p* < 0.05). Most other amino acids, with the exception of glycine and phenylalanine, also showed a negative response to increased *p*CO_2_ levels.

**Figure 4:**
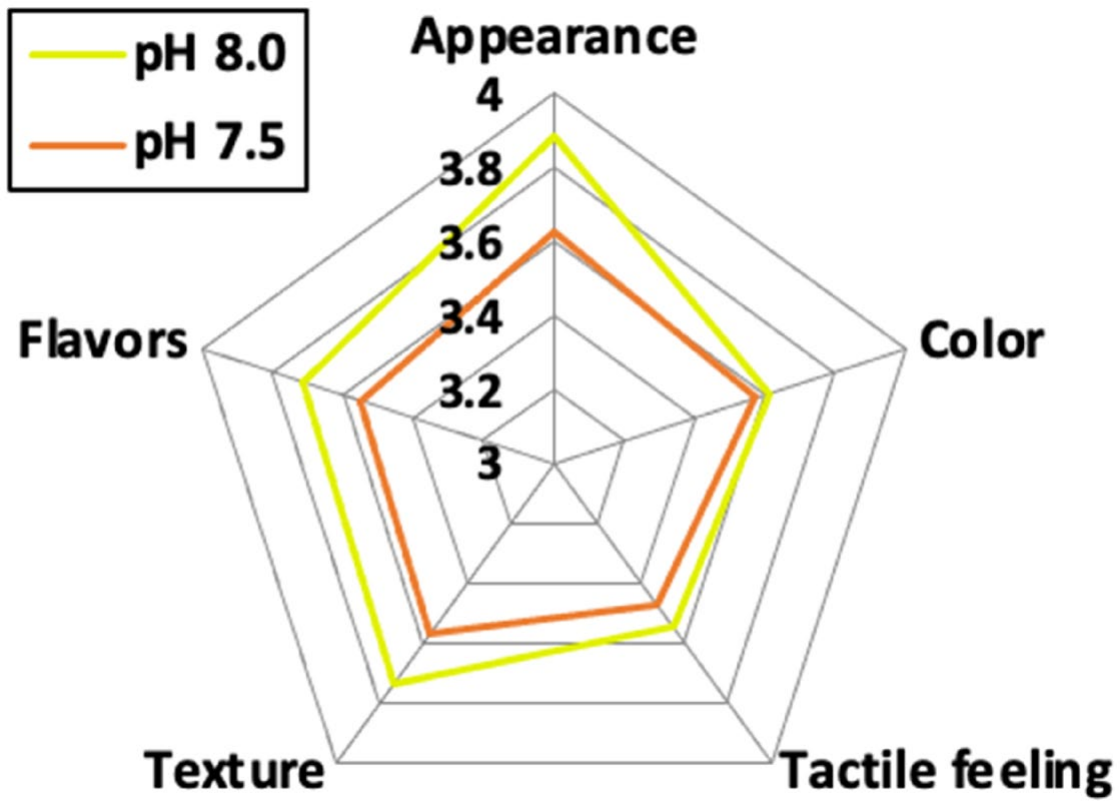
Average scores of appearances, color, tactile feeling, texture and flavor from two different pH treatments (8.0 and 7.5).

Sensory testing, which involved 40 test participants, yielded a similar score for the color and tactile perception of shrimp reared at pH 8.0 and pH 7.5 (Fig. 4). On the other hand, participants gave higher scores for appearance, texture and flavor at pH 8.0 than at pH 7.5 (Fig. 4). Despite their semi-quantitative nature, these ratings are consistent with elevated levels of those amino acids which produce the flavors preferred by consumers. The sum of amino acids (glutamate and aspartic acid) with flavors corresponding to umami (savory) was significantly higher at pH 8 than at pH 7.5 (n = 6, *p* = 0.03), but the amino acids responsible for sweetness (n = 6, *p* = 0.31) and bitterness (n = 6, *p* = 0.15) did not show differences between two pH exposure group (Table 1). The production of amino acids representing the umami flavor showed a significant pH dependence (*F* = 5.622, *p* = 0.045). The concentrations of total amino acids and the amino acid representing sweetness and bitterness were not affected by pH or salinity (Table 2).

**Table 1:** Temperature, salinity, pH, *p*CO_2_, saturation state for calcite (Ω_calcite_) and aragonite (Ω_aragonite_), total amino acids (TAAs) in tiger shrimp, and total amino acid concentration in each of the three taste classes at pH 8.0 and 7.5.

	pH 8.0	pH 7.5
Temperature (°C)	25.9 ± 0.13	26.0 ± 0.09
Salinity	32.09 ± 0.09	32.11 ± 0.08
pH	7.96 ± 0.005^a^	7.51 ± 0.07^b^
*p*CO_2_ (µatm)	543 ± 6.04^a^	1273 ± 10.0^b^
Ω_calcite_	4.37 ± 0.12^a^	1.46 ± 0.03^b^
Ω_aragonite_	2.87 ± 0.08^a^	1.0 ± 0.02^b^
TAAs (g 100 g^−1^)	19.5 ± 0.69^a^	17.65 ± 0.13^a^
Sweet taste (g 100 g^−1^)	4.64 ± 0.20^a^	4.42 ± 0.06^a^
Umami taste (g 100 g^−1^)	4.97 ± 0.22^a^	4.39 ± 0.04^b^
Bitter taste (g 100 g^−1^)	7.12 ± 0.26^a^	6.70 ± 0.08^a^

**Table 2:** Two-way ANOVA for the effects of salinity and pH on total amino acid expression and the different types of flavors they elicited.

	df	SS	MS	F	*p*
**pH**					
Total amino acids	1	5.913	5.913	3.242	0.109
Sweetness	1	0.148	0.148	1.182	0.309
Umami	1	1.027	1.027	5.622	0.045*
Bitterness	1	0.521	0.521	2.332	0.165
**Salinity**					
Total amino acids	1	0.066	0.066	0.036	0.854
Sweetness	1	0.012	0.012	0.099	0.761
Umami	1	0.008	0.008	0.046	0.836
Bitterness	1	0.029	0.029	0.129	0.729
**pH** × **Salinity**					
Total amino acids	1	0.139	0.139	0.076	0.789
Sweetness	1	0.222	0.222	1.764	0.221
Umami	1	0.029	0.029	0.160	0.699
Bitterness	1	0.402	0.402	1.799	0.217

df = degree of freedom, SS = sum of squares, MS = mean squares, F = value of F statistic, *p* = *p*-value. Significant difference at *p* < 0.05 is indicated by asterisk.

## Discussion

CO_2_ induced acidification represents a serious perturbation of the carbonate system. Most prawn have strong ion regulation ability in acid–base homeostasis^20^ while shrimp tend to enhance calcification rate in their shell in response to elevated *p*CO_2_^21,22^. In general, *P. monodon* will complete a molting cycle in 6 to 12 days^23^. Inhibition of molting in low pH cultures has been reported in previous studies on shrimp (*Lysmata californica*)^24^, where it actually resulted in a thicker cuticle, and this was also observed in other decapods^25^. Other decapods studies have also shown that their feed conversion rate, growth rate, and survival rate decreased significantly when pH dropped significantly below 8.0^26–28^. Even short-term exposure of crustaceans to low pH may induce dissolution in their exoskeletal CaCO_3_ in an attempt to buffer protons and maintain the homeostasis of the hemolymph^29,30^. The energy cost for shrimp to maintain pH homeostasis can be anticipated to increase as a consequence of OA^22,31^. This energetic burden of acidification is also likely to lead to higher mortality in shrimp exposed to lower pH.

In this study we found a clear decline in the concentrations of several amino acids in the flesh of shrimp upon exposure to low pH. This may have been related to the high concentration of CO_2_ which altered the chemical composition of seawater, potentially limiting the ability of the Na^+^/K^+^ pump to maintain the cell’s transmembrane potential and restricting the role of transport/detoxification proteins^34,35^. Previous studies have shown that the expression of antioxidant proteins and mRNA were decreased while shrimp was under acid stress. Similarly, a notable suppression of protein and carbohydrate digestion and absorption pathways was reported in low pH water^36^. In view of these findings, it can be speculated that the higher energy expended by the shrimp to maintain metabolism under conditions of pH stress may have led to the observed low concentrations of several amino acids present in the shrimp flesh.

To our knowledge, this is the first evidence of a possible link among (1) seawater pH, (2) the amino acid contents in tiger shrimp flesh and (iii) the sensory properties of the shrimps. The expression of amino acids can potentially be affected by salinity^37,38^, and thus affect the flavor of tiger shrimp. Here we find that elevated CO2 levels depress the production of the amino acid responsible for the umami flavor (Table 2). This is not altogether surprising given that amino acids are commonly found in artificial seafood flavorings. In nature, catabolic pathways of amino acids involve a series of decarboxylation, transamination and deamination reactions producing carbohydrates, alcohols, aldehydes and carboxylic acids that add extra flavor to shrimp^39^. Amino acids in shrimp flesh provided strong evidence to observe the impact of acidified water on tiger shrimp. Sulphur amino acids (methionine and cysteine) play important roles on oxidative stress resistant enzyme expression. While invertebrate expose to acidic water, higher oxidative stress (superoxide, reactive oxygen species) was detected in their tissue^40,41^. The decreased of methionine observed in our study, results in lower methionine metabolism affect low expression of cysteine^42,43^. Metabolic failure in a series of sulphur-containing amino acids could reduce antioxidative stress in tiger shrimp in acidified environment. From a consumer perspective, these decreased amino acids reduced the nutrient value of the shrimp.

Results of this study are very compelling and provide insights into the possible changes in amino acids and corresponding tastiness of the flesh and alteration of exoskeleton structure of tiger shrimp under future ocean acidification. However, we cannot extend these observed changes to other shrimp species due to species-specific responses of marine organisms to ocean acidification. For instance, increased calcification was observed in the exoskeleton of *Lysmata californica* (red rock shrimp)^24^, while calcification was unchanged in *Hippolyte californiensis* (California grass shrimp)^31^. On the other hand, growth rates were not altered in most of the shrimp species studied to date at low pH conditions^24,27,31,44^. Considering such limitations, we strongly recommend conducting extensive studies on the impacts of ocean acidification on amino acid compositions and tastiness of other shrimps especially, commercially important species.

The first two factors that customers consider when choosing shrimp are appearance and flavor. As ocean acidification intensifies, our results suggest that both qualities may be adversely affected. A reduction in customers satisfaction with shrimps cultured or harvested under ocean acidification conditions will potentially affect the global seafood aquaculture industry. For instance, the global trade of tiger shrimp, which is currently worth US$10 billion, will likely decline if shrimp look and taste worse. On the other hand, if our observations are limited only to specific shrimp species, it is possible that some other species may find a way to adapt to the projected higher acidity that would benefit both the shrimp and the shrimp industry. Under such circumstances, intensive culturing of species like tiger shrimps may be decreased due to the expected drop in demand, while more adaptive species may become popular among farmers. Hence, it is even possible that OA may drive the world aquaculture industry to culture different species from those presently favored.

The wild tiger shrimp is widely distributed around the Indian and western Pacific Ocean and is an invasive species in the Gulf of Mexico and along the Atlantic coast of the SE United states. It spends its early life history as larvae in estuarine environments before moving to deeper (~ 25 m) shelf waters where it starts its growth as young adult. The widely fluctuating environmental conditions in estuaries mean that tiger shrimp are reasonably well adapted to changes in salinity, pH and other hydrochemical variables, albeit during their early life history^45^. When shrimp move to deeper water, they encounter much smaller variations in the ambient pH. For our culture experiments, the commercial supplier provided us with tiger shrimp larvae kept in low-salinity medium (S = 5) which we then adjusted slowly till the salinity was the same as in the nearby coastal waters. The precise value of pH was more difficult to control on account of the many factors that affect pH, not least photosynthesis in phytoplankton and microbial respiration of organic matter, and which resulted in some very large short-term pH fluctuations (7.57–8.63) in our pH 7.5 culture tanks. This study and others report the potential effect of OA on marine organisms’ physiology, morphology, etc., but it should also be kept in mind that OA may also affect the generation time or reproduction^51,52^ of entire communities. It should also be noted that the exposure time in our experiment was relatively short compared to the adult tiger shrimp’s lifespan. Nevertheless, our results indicate a clear connection between acidification and the amino acid profile of the shrimp and their survival.

It is worth mentioning that OA would not significantly affect survival rate of adult white shrimp (*Litopenaeus vannamei*) at pH 7.5 as compared to white shrimp reared at pH 8.0^46^. If this is the case, the influence of OA on tiger shrimp may be greater than on white shrimp in the USA. Given that tiger shrimp are not native to North American waters, OA may mediate the success of this invasive species in the coastal region of Atlantic Ocean and Gulf of Mexico^47^.

## Materials and methods

### Acclimation of shrimp and exposure experiment design

Tiger shrimp (*Penaeus monodon*) were obtained as larvae and cultivated for 11 months at the shrimp pond at National Sun Yat-Sen University, Kaohsiung, Taiwan, in 2019. The pH manipulation experiment was started on March 25th, 2020. The time course of salinity and pH variations during shrimp cultivation periods is shown in Figure S1. Individuals with 19.7 ± 1.4 cm body length and 41.8 ± 10.2 g body weight were transferred into four 1000-L Fiber Reinforced Polymer (FRP) tanks which received an input flow rate of coastal water of 11 L min^−1^ each and were comprised of two replicated tanks per pH treatment. There were 60 shrimp per tank, which amounted to a stocking density as 53 shrimp per m^2^. Tiger shrimp were acclimated to tank conditions for a week and then they were kept at 26–28 °C, S = 30–32 psu, and fed 5% of shrimp weight of commercial pellets four times every day. Once a day, the tanks were cleared of uneaten feed and detritus that had settled to the bottom.

After a week of acclimation, shrimp belonging to the high pH group were maintained in natural flowing coastal seawater at pH ~ 8.0 (no added CO_2_) and pH was measured once a day (WTW ProfiLine pH 3110, accuracy ± 0.005). Shrimp in the acidified tanks were exposed to a gradually decreasing pH from 8.0 to 7.5 (decreasing by 0.1 per day) using an automatic pH feedback system (P-LE-08 Digital pH controller, Leilih). Dead shrimp and uneaten feed were removed from the experiment tanks before providing each new feed. The percent survival of tiger shrimp is an average number in pH 8.0 and 7.5. The equation was described as follow:

$$Percent \;survival = n_{initial} - n_{dead} /n_{initial}$$

n_initial_ is the initial number of shrimp and n_dead_ is number of dead shrimp in daily. Sensors belonging to both pH controller and WTW meter were calibrated using NIST standard buffers having pH values of 4.01, 7.00 and 10.00 (± 0.02 at 25 °C). Seawater *p*CO_2_ was continuously measured using the air-gas equilibration technique, associated with K30 CO_2_ sensor (accuracy ± 30 ppm). CO_2_ sensor was calibrated against standards CO_2_ gases of 600, 3000 and 6000 ppm. Concentrations of CO_3_^2−^ and calcium carbonate saturation states were calculated using the CO_2_ SYS computer program^48^ using measured pH and *p*CO_2_ as the input carbonate system parameters with dissociation constants (K1 and K2) from Dickson and Millero^49^.

### Thickness and carbon content of shrimp cuticle

After 28 days exposure, shrimp carapace thickness was measured with a 0.05-mm accuracy using a thickness gauge (AICE, China). The carapace was then washed with deionized water to remove large particles, and then ultrasonicated in 1 N NaOH for 10 min to remove proteins and pigments. The washed cuticle was dried and homogenized into power for measuring total carbon (TC) and particulate organic carbon (POC) by elemental analysis (Elementar vario EL cube, Germany). The particulate inorganic carbon (PIC) content in the cuticle was estimated as the difference between TC and POC.

### Amino acids extraction and measurements

At the end of the 28-day exposure experiment, three shrimp from each tank were sampled for analysis of free amino acid content. Subsamples of 0.05 g of shrimp flesh were processed by AOAC method^50^, digested into 1 mL of 6 N HCl, then dried and reconstituted in 1 mL of 0.02 N HCl. The digested samples were filtered through a 0.2 µm Supro membrane disc filter. Total amino acids were measured and quantified in these extracts using an Amino Acid Analyzer L-8900 (Hitachi, Japan).

### Evaluation of shrimp sensory quality under pH-8.0 and pH-7.5 treatments

After 28 days exposure, forty shrimp from each tank were harvested and soaked in icy cold seawater for a few minutes until they died. These shrimp were immediately cooked in boiling seawater (salinity = 30) for 3 min. After natural cooling, a blind test of the shrimp sensory quality was carried out by 40 volunteer assessors consisting of 23 males and 17 females from 7 countries. All of them liked to eat shrimp and they had eaten shrimp more than 5 times within the previous 6 months. Shrimp from each experimental group was randomly served to the participants. In brief, four shrimp (two from pH 7.5 and two from pH 8.0) were put in four different plates individually and sent to each participant randomly (Fig. S2). The exact nature of the study was withheld from the participants who were simply asked to score the four shrimp (two shrimp for each pH 8.0 and 7.5) based on their appearance, color, tactile feeling, texture and flavor. For each category, they gave a score to the shrimp specimen from 1 (worse) to 5 (best).

### Statistical analysis

The gustatory sensation of amino acids in tiger shrimp was calculated by summing up all the amino acids known to elicit a specific taste. As such, the amino acids were divided into three classes. Glycine, alanine, threonine, proline, serine and glutamine contributed to sweetness. Glutamate and aspartic acid delivered the umami taste, while the bitter sensation came from phenylalanine, tyrosine, arginine, leucine, isoleucine, valine, methionine and histidine^16–19^.

Statistical analyses were performed by using IBM SPSS software (version 24.0). The statistical significance level of 0.05 was chosen for all statistical tests. Normality of all data were checked using the Shapiro–Wilk normality test, prior to any other statistical analyses. A linear regression was used to estimate the survival rate in different treatments of pH in tiger shrimp. Student’s t test was conducted to decide whether there were any significant differences in water chemistry, thickness and carbon content of shrimp cuticles and amino acid contents of shrimp flesh under the two different pH exposures. Two-way ANOVA were used to examine the combined effects of salinity and pH and their interactive effects on the expression of total amino acids and on the different flavors provided by the amino acids.

## Supplementary Information

Supplementary Information.

## Data Availability

All data are available in the main text or the supplementary materials.
