# Supplementary material for: The effects of low pH on the taste and amino acid composition of tiger shrimp
Source: Sci Rep. 2021 Oct 27;11:21180. doi: 10.1038/s41598-021-00612-z (PMC8551290; doi:10.1038/s41598-021-00612-z)
Supplement: Supplementary file 1 — Supplementary Information. [file 41598_2021_612_MOESM1_ESM.pdf]

The effects of low pH on the taste and amino acid composition of tiger shrimp

Hsueh-Han Hsieh[1], Veran Weerathunga[1], W. Sanjaya Weerakkody[1,2], Wei-Jen Huang[1], François L. L. Muller[1], Mark C. Benfield[3] and Chin-Chang Hung[1,*]

1. Department of Oceanography, National Sun Yat-sen University, Kaohsiung, Taiwan, R.O.C.
2. Department of Fisheries and Aquaculture, Faculty of Fisheries and Marine Sciences and Technology, University of Ruhuna, Matara, Sri Lanka
3. Department of Oceanography and Coastal Sciences, Louisiana State University, LA, US.

*Corresponding author. Email: cchung@mail.nsysu.edu.tw

This PDF file includes:

Fig. S1

Fig. S2

Tables S1

Tables S2

Table S3

Fig. S1.

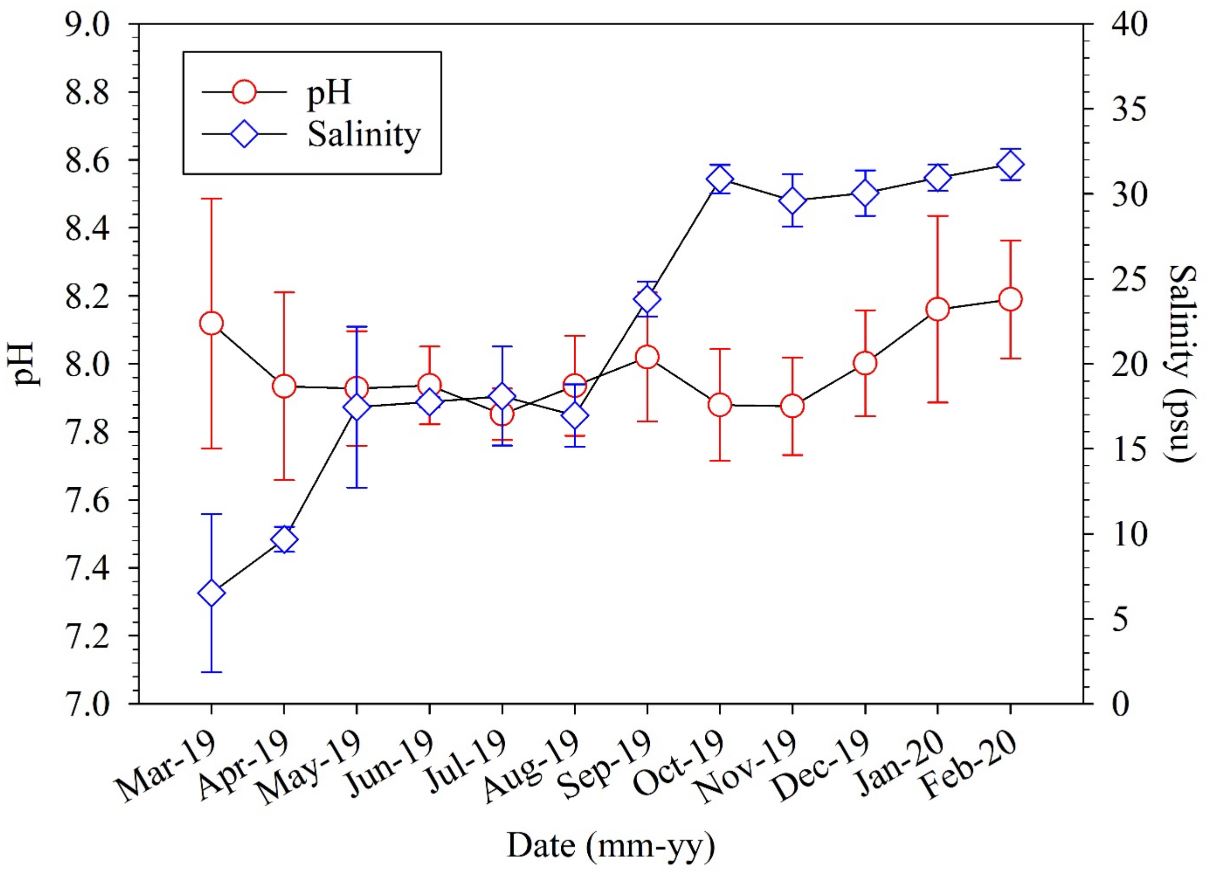

Fig. S1. Salinity and pH in acclimation state. Red circle: pH, blue diamond: salinity (mean ± S.E).

Fig. S2.

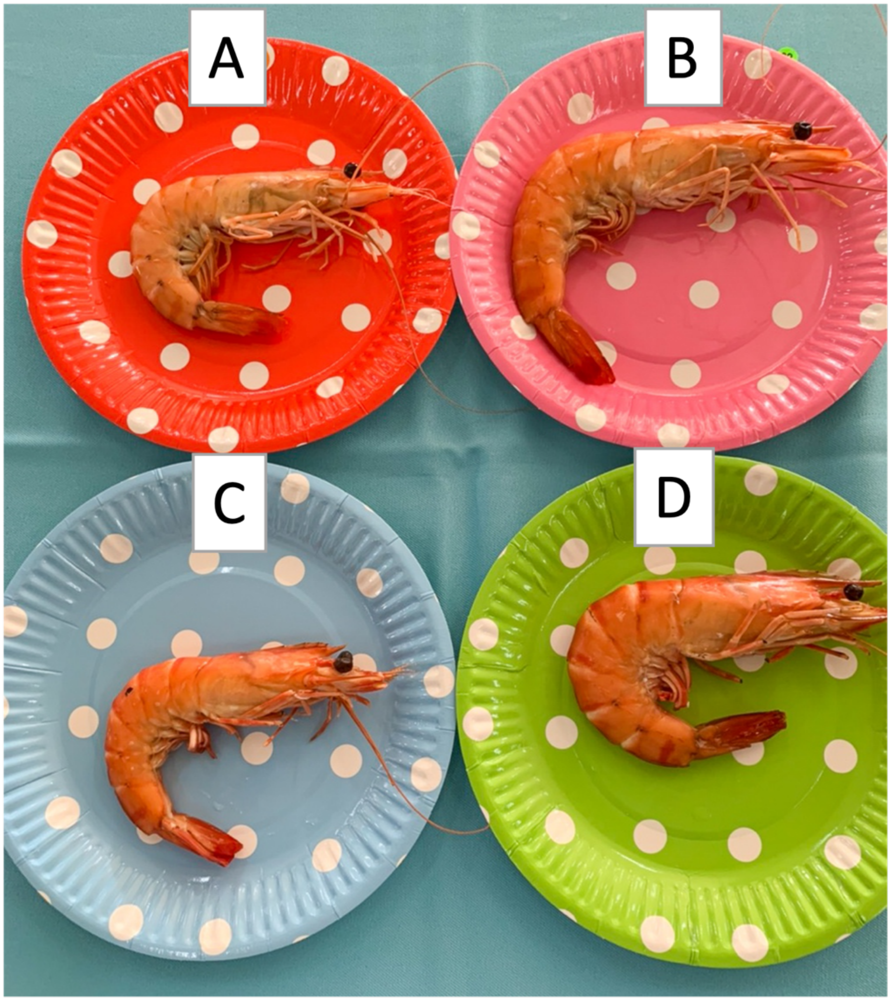

Fig. S2. The shrimp in pH 8.0 was in plate (A) and (D), in pH 7.5 was in plate (B) and (C).

Table S1.

Table S1. The composition of total amino acid (g $100g^{-1}$) in tiger shrimp muscle.

	pH 8.0 (g/100 g protein)	pH 7.5 (g/100 g protein)	Peñaflorida (1989) (g/100 g protein)	Sriket (2007) (g/100 g protein)
Aspartic acid	10.88	9.71	8.83	8.59
Threonine	4.24	3.71	3.23	7.24
Serine	4.29	3.71	3	6.29
Glutamic acid	18.35	16.12	13.96	10.88
Glycine	12.24	12.76	4.9	6.94
Alanine	6.53	5.76	4.95	9.00
Cysteine	2.47	1.65	0.63	3.12
Valine	5.41	4.53	4.21	6.82
Methionine	2.29	2.06	2.3	8.18
Iso-leucine	4.47	3.94	3.89	15.24
Leucine	7.35	7.12	6.61	17.47
Tyrosine	3.65	3.65	3.24	11.47
Phenylalanine	5.06	5.76	3.2	13.35
Lysine	9.24	9.00	6.8	3.82
Histidine	2.29	2.12	1.91	3.94
Arginine	11.35	10.24	8.28	25.12

Table S2. Analysis of variance where the sources of variation are due to pH, salinity or pCO_2 treatments. MS = mean squares, F = value of F statistic, p = p-value.

		Model	MS	F	P
pH	pH 8.0	$F_{1,52}$	0.0014	1.4390	0.2350
	pH 7.5	$F_{1,52}$	0.0017	1.0160	0.3180
	pH 8.0 vs 7.5	$F_{2,77}$	1.7430	1572.42	<0.001
Salinity	pH 8.0	$F_{1,52}$	0.0003	0.0012	0.9726
	pH 7.5	$F_{1,52}$	0.0474	0.3034	0.5841
	pH 8.0 vs 7.5	$F_{2,77}$	0.0258	0.2463	0.7829
pCO2	pH 8.0 vs 7.5	$F_{1,12}$	1839687.5	1230.3055	<0.01

Table S3. P-value of statistical analysis of sensory testing between local nationality and others nationalities.

	Appearance	Color	Tactile feeling	Texture	Flavor
pH 8.0	0.86	0.4	0.92	0.12	0.09
pH 7.5	0.16	0.63	0.51	0.92	0.92

Reference

1. Peñaflorida, V. D. An evaluation of indigenous protein sources as potential component in the diet formulation for tiger prawn, *Penaeus monodon*, using essential amino acid index (EAAI). *Aquaculture*. **83**, 319-330. (1989)
2. Sriket, P., Benjakul, S., Visessanguan, W., Kijroongrojana, K. Comparative studies on chemical composition and thermal properties of black tiger shrimp (Penaeus monodon) and white shrimp (Penaeus vannamei) meats. *Food Chem.* **103**, 1199-1207 (2007).
